# A fully automated framework for acoustic identification and localization of terrestrial wildlife at scale

**DOI:** 10.1038/s42003-026-09949-5

**Published:** 2026-05-09

**Authors:** Louis Freeland-Haynes, Sam Lapp, Chapin Czarnecki, Lauren Chronister, Joe Begley, Alex Rogers, Peter Prince, Andy Hill, Justin Kitzes

**Affiliations:** 1 University of Pittsburgh, Pittsburgh, PA USA; 2 Computer Science, University of Oxford, Oxford, UK; 3 Open Acoustic Devices, Southampton, UK

**Keywords:** Conservation biology, Machine learning, Behavioural ecology

## Abstract

Autonomous acoustic recorders are increasingly important tools for monitoring sound-producing taxa. Coupled with advances in machine learning, they enable greater sampling effort and larger spatial and temporal scales than in-person surveys. With synchronized recorders, acoustic localization enables collection of fine-grained spatial data on animals’ positions. Spatial data has important applications in abundance estimation, territory delineation, studies of movement and microhabitat use. Applications of acoustic localization have however been limited in scale, due to the technical and cost challenges of hardware and effort-intensive analysis steps.. We present a fully automated framework for identification and acoustic localization of terrestrial wildlife. Using low-cost GPS-Audiomoth recorders, we provide an open source software pipeline for detection, time-delay estimation, localization, error rejection and resolution of multiple simultaneous sound-sources. We achieve high spatial accuracy on a loudspeaker test, where 99% of broadcast calls localized were accurate to within 5 m. We demonstrate its utility for surveying birds at large spatial scales with a localization array of over 60 recorders at a forested site. Using a CNNfor automated detection, our localization framework produces spatial patterns of species observations similar to those found by in-person spot mapping surveys. Our framework enables applications of acoustic localization at large scales.

## Abstract (web summary)

Automated pipeline detects species calls and localizes animals from continuous audio, reducing manual review and helping to scale wildlife monitoring across environments.

## Introduction

Advances in autonomous sensor technologies and methods for analyzing the data collected by these sensors are in the process of transforming the nature and scale of ecological data. In the case of sound-producing taxa, acoustic monitoring with autonomous recording units (ARUs) offers significant advantages over traditional monitoring methods: acoustic monitoring is minimally invasive, the vastly increased sampling intensity enables monitoring of rare events and taxa, and the data collected can serve as acoustic specimens that can be reanalyzed as new analysis techniques are developed or to answer new questions^1^. In terrestrial ecosystems, a diverse array of taxa, including insects^2^, bats^3^, mammals^4^, and birds, are well-suited to acoustic monitoring, and acoustic monitoring has been shown to outperform human-based surveys for surveying avian communities at large spatial and temporal scales^5^.

As advances in recording hardware like the open source AudioMoth^6^ have made acoustic monitoring increasingly accessible and affordable, the development of methods for extracting rich biological information from acoustic data has become key. There is a strong need for increasingly automated workflows that can reduce the time and cost associated with the more effort-intensive steps of analysis, to enable ecologists and land managers to take advantage of the potential benefits in scale that acoustic monitoring offers. Automated sound detection and identification, particularly deep-learning-based methods^7^, hold great promise for reducing the amount of human listening effort required for ecological inference from acoustic data. For avian communities, deep learning models capable of identifying many species globally are now freely available^8,9^ and are beginning to be used for ecological research^10^.

Although automated passive acoustic monitoring can collect vastly more data than possible with in-person surveys^11^, there are some important limitations to the types of data typically collected by large scale acoustic deployments. One of the primary limitations, compared to the data possible with in-person surveyors, is in the spatial resolution of detections of an animal. In a recording from a single microphone, the only spatial information that can readily be inferred from an acoustic detection is that the animal was present within some detection distance of the recorder. Previous attempts to address this challenge include using calibration data to estimate the effective detection radius of an acoustic recorder, which may be used to standardize in-person surveys and acoustic recordings^12^. Similarly, with calibrated recordings, the sound level of a vocalization provides an estimate of the distance from the recorder, enabling distance-sampling based density estimation^13^. Other methods include cue counting and vocal density estimation, see in ref. ^14^ for a review. Despite these advances, the absence of finer-grained spatial information, such as 2D or 3D co-ordinates of the locations of individual animals, makes it challenging to apply the increases in scale made possible by bioacoustics to the investigation of ecological questions of behavior and movement, microhabitat use, or to use distance sampling methods^15^ to estimate true abundance and density.

Acoustic localization is a promising method for collecting fine-grained spatial data on the positions of animals for enabling abundance estimation and the investigation of movement and behavior. By deploying arrays of microphones (localization arrays) capable of recording in a time-synchronized manner, it is possible to estimate the location of a sound source from the relative arrival times of the sound at multiple recorders. Within acoustic monitoring, acoustic localization arrays have seen extensive use in marine systems where they enable the collection of behavioral data on otherwise hard to observe organisms like deep-diving cetaceans^16^. In terrestrial systems, acoustic localization has been used for a variety of ecological applications, and reviews of both methods and applications can be found in refs. ^17,18^. Applications in terrestrial systems have included tracking bird display flight paths^19^, measuring inter-individual distances in chorusing frogs^20^, quantifying sonar beam focus in hunting bats^21^, estimating heights of nocturnal migrant birds^22^, investigating bird microhabitat choice^23^, localizing howling wolves over large distances^24^, and estimating animal abundance^25,26^.

Though acoustic localization can be used to collect spatial information on the locations of animals, most existing applications have been limited in their scale. In order to use time difference of arrival (TDOA) based methods for localization, audio recordings from multiple recorders must be time-synchronized, which, depending on the spatial scale of the array and spatial precision demanded may require sub-millisecond level synchronization. Tightly time-synchronized recordings are most easily achieved with cabled rather than fully autonomous recorders, which necessarily limits the spatial scale of cabled localization arrays e.g.,^27,28^. Additionally, many of the downstream analysis steps involved in identifying sound-source locations from time-synchronized audio are effort-intensive when performed manually: listening to and annotating the audio for vocalizations of interest, measuring the time differences of arrival of a vocalization between different recorders, and estimating the sound source location and accuracy of localization given those time differences. To apply acoustic localization to larger spatial and temporal scales requires uncabled time-synchronized recording hardware and greater automation of the acoustic localization framework.

Here, we present an automated time difference of arrival based localization framework for acoustic localization of terrestrial animals using autonomous recording units. By pairing an automated detection method for detecting vocalizations with automated time-delay estimation, sound source localization, and error rejection steps, this framework can be used to estimate coordinate positions of vocalizing animals within an array of recorders in a fully automated fashion. We show through a playback test with speakers broadcasting bird vocalizations that the framework is able to accurately localize sounds, and through a field deployment at a forested site demonstrate its utility for collecting fine-grain spatial data on an avian community. We compare the positions of singing birds observed with our framework to the spatial patterns of observations obtained with traditional in-person spot mapping surveys. Our results demonstrate that this framework recapitulates spatial patterns found by traditional surveying even in a dense soundscape, and enables the autonomous collection of vastly more spatio-temporal observations than in-person surveys.

## Methods

### Localization framework

**Fig. 1:**
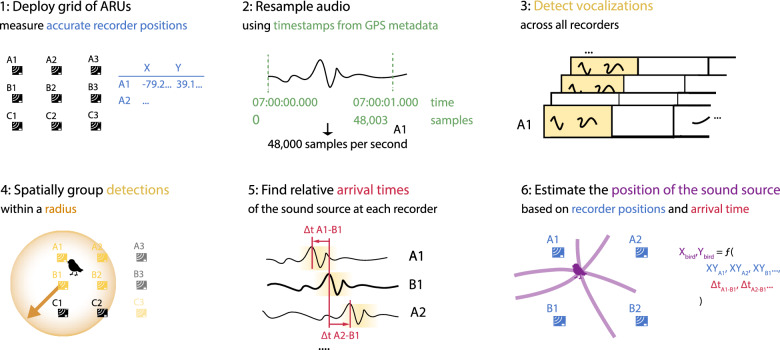
An overview of the steps involved in the automated localization framework. In step 1, synchronized audio is collected from recorders at known positions. In step 2, GPS timestamps are used to resample the audio so that recordings from all of the autonomous recorders are time-synchronized. In step 3, an automated detection method is used to detect sounds of interest. In step 4 detections on nearby recorders are grouped together, leading to multiple partially redundant “candidate sets” of recorders likely to contain the same vocalization. In steps 5–6, time differences of arrival are automatically estimated and position estimates produced for each “candidate set”. In step 7, the position estimates from these multiple candidate sets are clustered. Error rejection filters occur after steps 3, 5, and 6.

Our automated detection and localization framework involves a number of consecutive steps, which are outlined in Fig. 1. The steps consist of:

1. Deploying recorders to collect audio. We use recorders that receive GPS timestamp information that enables time-synchronization across multiple autonomous recorders. The coordinate positions of these recorders are also precisely measured with GPS receivers during deployment.
2. Post-processing the audio using GPS-received timestamp data to produce synchronized audio files.
3. Detecting and identifying vocalizations in the synchronized audio using an automated detection method.
4. Identifying “candidate sets” of recorders that are likely to contain detections of the same vocalization.
5. Estimating relative arrival time of the vocalization at each receiver, for each candidate set.
6. Estimating a sound source location from the relative arrival times and microphone positions, for each candidate set.
7. Identifying sound source locations by clustering redundant location estimates from candidate sets that originate from the same time windows.

We apply filtering criteria throughout this framework to remove candidate sets with low accuracy or self-consistency. This section will proceed with an overview of each step in the framework, with details of the parameter choices that can be made by a user at each step. These parameters are also given in Table 1. For clarity, in addition to describing these choices, we also provide the names of variables used to specify them in OpenSoundscape v0.10.2^29^, the open source python package that implements this framework. We point users to the OpenSoundscape documentation (www.opensoundscape.org) for further documentation and a tutorial on acoustic localization that will be kept up to date with changes in the package.

**Table 1:** Parameters for the localization framework

Parameter	Parameter name in OpenSoundscape v0.10.2	Parameter options	Value chosen in experiments	Rationale
Time-window length for automated detection and localization		Any time length	4 s	Must be longer than the maximum possible time delay, which corresponds to the furthest distance in the array over which the same sound-event can be detected. Should also be short enough that the vocalization is likely to make up a significant portion of the audio signal for accurate time delay estimation.
Cross-correlation filter	“cc_filter”	Normalized, Phase transform, Roth transform	Phase Transform	The phase transform was shown to work well in the case of bird song localization in a simulated forest soundscape^33^.
Cross-correlation threshold	“cc_threshold”	Between 0 and 1	0.01	TDOAs that are spurious tend to have a low cross-correlation value. To choose this value we inspected a histogram of cross-correlation values from random audio recorded during the speaker test (i.e. uncorrelated noise signals) and picked approximately the mean cross-correlation value. Note: the cross-correlation score reflects signal similarity after bandpassing and applying the cross-correlation filter, which means the values of these scores will change depending on the filter used.
Minimum number of recorders to attempt localization	“min__receivers”	Integers > *n* + 1 Where *n* is the number of dimensions for localization	4	The minimum number necessary for localization in 2 dimensions.
Maximum detection distance	“max_receiver_dist”	Distances >0 m	150 m	Estimate of the maximum distance over which it is likely that 2 detections are of the same sound-event.
Bandpass Range	“bandpass_ranges”	Any frequency range	A set of species-specific bandpass ranges, chosen by evaluating recordings.	Reduces the effects of non-target noise on TDOA estimation. We chose frequency ranges that centered on the majority of a vocalization’s energy.
Localization Algorithm	“localization_algorithm”	Least squares optimization of the TDOA residuals, Bancroft GPS algorithm^35^, Gillette^49^	Bancroft GPS algorithm	Used in past bioacoustic localization frameworks^28^.
TDOA residual RMS threshold	“residual_rms_threshold”	Any real number > 0	0.0146 s	A measure of the error in the observed TDOAs vs expected TDOAs. We chose a time value that represents a 5 meters root mean square error given the speed of sound (343 m/s).
DBScan Epsilon		Distance > 0 m	5 m	Epsilon is the maximum distance between two points for them to be considered neighbors.
DBscan minPoints		Integers ≥ 2	3	One greater than the minimum number of points required by DBScan for clustering.

### Hardware and audio synchronization

Estimating time differences of arrival (TDOAs) for a sound detected on multiple receivers requires audio from these receivers to be synchronized. In this context, audio synchronization means that the timing of audio samples across multiple devices is closely aligned. For instance, if two recorders started recording at exactly the same time, and recorded at exactly the same sample rate, they would produce synchronized recordings. In practice, the internal clocks of autonomous recorders typically drift over time such that they gradually deviate from tracking the correct time, and the internal oscillators exhibit variations such that they do not consistently sample at the nominal sample rate.

Both problems can be addressed by using a recorder equipped with a GPS receiver. For our framework, we use open source AudioMoth field recorders^6^ that receive NMEA RMC format messages and pulse per second (PPS) signals from a connected GPS receiver. The AudioMoth writes GPS timestamps decoded from NMEA RMC messages, and the number and offset of audio samples relative to each GPS PPS signal, within a metadata file (see Supplementary Table 1). During post-processing, this metadata is used to generate precisely timed and resampled audio recordings that match the nominal sample rate. This procedure accounts for differences in clock time and sampling rate across different recorders, and also corrects for any drift in a device’s sampling rate throughout the recording period.

### Automated detection and identification of sounds

After synchronizing the audio, the framework begins with the automated detection of vocalizations of interest. We detect vocalizations of interest in discrete time-windows of synchronized audio (Fig. 1 Panel 3). We use a custom convolutional neural network (CNN) trained to identify species-specific birdsong in spectrograms, visual representations of a time-window of audio. CNNs produce a continuous score output for each class of sound the model has been trained to identify, which represents the model’s confidence that a species’ song is present in that discrete time-window. To turn these continuous scores into binary (1/0) detections, we apply thresholds, converting each score above the threshold to a 1 and each score below the threshold to a 0. Though we use a CNN, any detection procedure that produces binary detections for the presence of one or more sound classes in discrete time-windows can be used. Each sound class corresponds to a category of sound of interest, for instance a species or vocalization type.

The detection process produces binary 1/0 detections for discrete time-windows, for every recorder within the array. The length of the time-windows used for detection is an important design choice. In our applications detecting and localizing birdsong, we use 4 second time windows. Time delay estimation will be performed using audio that corresponds to the same time-window on multiple recorders. As a result, time windows must be longer than the maximum possible time difference, which corresponds to the furthest distance within the array over which the same sound-event could be detected. However, particularly in noisy environments, time windows should be short enough that the vocalization is likely to make up a significant portion (e.g. >⅓) of the time window, as this will decrease the likelihood of spurious non-target events dominating the audio and causing errors in time delay estimation. In many bioacoustic applications, pre-trained classifiers are likely already to provide appropriate window lengths. For example, BirdNET^9^ and Perch^8^ use 3 s and 5 s windows for detection, respectively, which are likely to be appropriate in most avian localization contexts.

### Identifying candidate sets of recorders that are likely to contain detections of the same sound-event

Estimating sound source positions relies on the detection of the same acoustic event, such as a bird’s vocalization, by microphones at different positions (Fig. 1 panel 4). At a given time point, detections of a species across the grid of microphones may correspond to the vocalizations of one or more individuals. We use spatial proximity of the recorders to identify subsets of recorders where the detections are likely to be of the same individual vocalizing. Specifically, we identify “candidate sets” of recorders using the following process. First, for each time-window (e.g., 10:00:00-10:00:04 Jan 1 2024), we identify all the recorders with detections of the same sound class. Each recorder will iteratively be considered a reference recorder. For each reference recorder, we create a “candidate set” consisting of the reference recorder and all other recorders within a user-specified radius (e.g., 100 m) that have a detection of the same sound class within the same time-window. For example, in Fig. 1 Panel 4, five recorders detected a vocalization at the current time window, and a candidate set is created from each of these five recorders (one such candidate set for Recorder B1 is illustrated). Depending on the geometry and distances between the recorders, these candidate sets may all contain the same set of recorders (but with a different central “reference recorder”) or may contain different subsets of the recorders. For each candidate set, we will estimate time delays of arrival at each recorder relative to the central reference recorder. For each candidate set, we then attempt localization from the TDOAs, as will be described in the next two sections.

This design of creating multiple candidate sets leads to multiple, partially redundant attempts at localization of the same sound-event. If multiple candidate sets produce similar location estimates, we can have higher confidence in the location estimate - a property which will be exploited by clustering of these position estimates later in the pipeline. If, however, a given candidate set is “poorly chosen” and actually contains recorders that have detections of different individuals, this can be caught by filtering steps that identify low signal similarity, contradictory TDOAs, and by the process of clustering which effectively discards estimates inconsistent with other candidate sets.

The maximum distance from the reference recorder for inclusion in a candidate set (“max_receiver_dist”, depicted as an orange arrow in Fig. 1 Panel 4) is an important parameter that must be specified. It represents the maximum distance over which detections on two recorders are likely to be of the same vocalization. This distance should be informed by biological knowledge, as the expected amplitude, frequency content of the vocalization, and acoustic properties of the habitat all affect the expected maximum detection distance for a sound^30,31^. The quality of the microphone on the recorders may also affect this distance. The expected temporal and spatial density of sound events, and the spatial density of recorders within the localization array, should also affect the choice of this distance. If sound-events are rare and expected to be found at low density, it may be appropriate to use a distance that is twice the maximum distance over which it is possible to detect a sound (e.g., “max_receiver_dist” = 200 m for a bird song that is detectable up to a maximum of 100 m from the sound-source). However, if vocalizing animals are expected to be found at high density in the array, a smaller distance may be preferable, as detections at greater distances are more likely to be of other individuals vocalizing. The minimum usable value for this distance will be dictated by the geometry and spacing of recorders within an array. For example, if recorders are on average >50 m apart, a “max_receiver_dist”= 50 m would be too low.

### Estimating time differences of arrival (TDOAs)

To estimate a sound source’s location, we first estimate time differences of arrival (TDOAs). These measure the relative arrival time of a sound at each recorder in a candidate set, relative to its arrival time at the reference recorder (Fig. 1 Panel 5). We do not estimate every possible pair-wise TDOA within a candidate set. We use the generalized cross-correlation algorithm (GCC)^32^ to automate estimation of time delays by finding the time-delay with maximal cross-correlation of the two audio signals. The time delay cannot be greater than the time it would take a sound to travel the maximum inter-recorder distance “max_receiver_dist”. Therefore, we estimate the TDOA by finding the time-delay with maximal cross-correlation within this bound.

The robustness of cross-correlation for time-delay estimation can be improved by applying spectral weighting functions. We used the phase transform filter (GCC-PHAT), as it is robust to reverberation and has been shown to perform well for estimating time-delays in simulations of avian forest soundscapes^33^. In our software, we expose the choice of spectral weighting function (“cc_filter”) as a parameter that may be chosen according to the sound type and acoustic context. To improve the accuracy of TDOA estimation, we also bandpass the audio to species-specific frequency ranges (“bandpass_ranges”) to reduce the effects of background noise. These frequency ranges can be chosen to cover the entire frequency range of a vocalization, or if many taxa overlap in frequency or the soundscape is particularly noisy it may be appropriate to more tightly limit the frequency range to the frequencies with greatest intensity for a vocalization.

In addition to the estimated time delay, generalized cross-correlation produces a cross-correlation value. This value represents the similarity of the filtered audio signals, when offset by the estimated time delay. This value can therefore be used as a “signal similarity score” to identify potentially erroneous TDOAs: a low “signal similarity score” suggests the two recorders do not contain similar audio signals and perhaps have detected different sound-events or that we otherwise have low confidence in the estimated TDOA. Our software implementation allows the user to optionally specify a minimum threshold for this signal similarity score (“cc_threshold”). In subsequent localization steps TDOAs from recorders with a cross-correlation value below this threshold will be dropped from a candidate set before localization is attempted.

The exact value for this parameter may be “tuned” by a user based on cross-correlation values found in audio from field data, as the optimal value will depend on the properties of the vocalization (its bandwidth, intensity, and length), the soundscape, and audio window-length used. We emphasize that this parameter is optional; however, a simple approach to setting its value is to inspect cross-correlation values of a randomly chosen subset of field audio. We suggest setting a relatively low value, in our case, we used 0.01, effectively discarding only TDOAs where the two filtered audio signals are highly dissimilar.

### Position estimation from a set of TDOAs

The previous section outlined how TDOAs are estimated automatically using generalized cross correlation. For each candidate set, TDOAs were estimated relative to the reference recorder. We now use those TDOAs to estimate a sound source location (Fig. 1 Panel 6). There are several algorithms for estimating a sound source location from relative times of arrival of a sound at a set of known positions. These include optimization approaches that minimize the time-delay residuals and closed-form approximations. Discussions of these approaches can be found in the positioning system literature^34^. We use a closed form algorithm that implements the Bancroft GPS method to solve a sound-source position from a set of TDOAs^35^. Our software implementation allows the user to select from localization algorithms listed in Table 1 (“localization_algorithm”). If a minimum “signal similarity” threshold as described above is specified, only TDOAs from recorders that exceed this “signal similarity” threshold will be used for localization.

Because the absolute time a vocalization was produced is unknown, detecting the vocalization on n receivers results in n-1 independent TDOA measurements. As a result, estimating a 2-dimensional position requires at least 3 recorders that are not all positioned on a line, and estimating a 3-dimensional position requires at least 4 recorders that are not all positioned on a plane. As localization with more recorders is expected to increase the confidence in a position estimate^18^, a user may optionally specify a larger minimum number of recorders (“min_n_receivers”) required in a candidate set for localization to be attempted. See Fig. 3 for a demonstration of how this affects accuracy and recall. Increasing the minimum may be useful if higher accuracy of position estimates is demanded and where the recorder array is densely placed, so that any sound event is expected to be detected on a higher number of recorders.

After producing a location estimate, we quantify how well that estimate matches the observed time delays. This is an important step in rejecting erroneous or poorly localized position estimates. We use the root-mean square of the time delay residuals for all the recorders in the candidate set as our error metric.

$${RMS}=\sqrt{\frac{1}{n}{\sum }_{i=1}^{n}{({t}_{i}-{\hat{t}}_{i})}^{2}}$$

Where t_i_ is the observed time delay between the central reference recorder and recorder_i_, and t_hat_i_ is the expected time delay given the distance between the position estimate, the recorder, and the speed of sound. Multiplying the root-mean-square of the residuals by the speed of sound results in a more easily interpreted error metric with units meters. In our software implementation, the user specifies a threshold for this error metric (“residual_rms_threshold”), and position estimates with higher error are discarded. The residual threshold chosen represents a minimum confidence in a localized position, and should be chosen based on the application—a lower threshold may be appropriate in applications that prioritize highly accurate location estimates over the total number of sounds localized. In our applications we used a “residual_rms_threshold” of 5 m, as we desired estimates with less than 5 m error.

### Clustering of position estimates

The framework, as described above, identifies candidate sets of recorders likely to have detected the same sound event, performs automatic TDOA estimation relative to the central recorder in that candidate set, and from those TDOAs estimates a sound source location. This results in multiple attempts at TDOA estimation and localization for a given sound event, up to one for each recorder that detected the sound event. The location estimates, therefore, represent multiple, partially redundant attempts at localization of a sound event. We expect accurately localized sounds to have multiple closely clustered position estimates, one for each candidate set where localization was successful. This provides another opportunity to reject low confidence estimated positions—by applying a clustering algorithm to identify clusters of position estimates (Fig. 1 Panel 7). We use the Density-Based Spatial Clustering of Applications with Noise (DBSCAN) algorithm, a density-based algorithm for identifying clusters in the presence of noise^36^ that does not require specifying the number of clusters. We apply this clustering to all position estimates for the same sound class, from the same time window. We then use the means of each cluster as the sound source location estimate. This clustering process also allows for the resolution of multiple simultaneous sound sources at different positions across the array, as distinct clusters of position estimates will be resolved.

DBScan requires the choice of two parameters, that at a high level can be understood as the maximum distance between two samples for them to be considered close neighbors, and the minimum number of samples for a point to be considered a core point. This clustering is implemented outside of the localization framework with the package scikit-learn^37^.

### Experiments

We tested our automated detection and localization framework in two separate experiments. In a loudspeaker playback test, we deployed loudspeakers at known locations within a 3 × 3 recorder microphone array and broadcast audio recordings of bird vocalizations. To apply the localization framework to a real avian community, we also conducted a field experiment, deploying a localization array of recorders at 61 points in a rectangular grid covering approximately 6.25 hectares at a forested site. To gather ground truth locations for birds in the field experiment, spot-mapping surveys by expert surveyors were also conducted at the site of the forest deployment.

### Loudspeaker experiment

In our loudspeaker experiment, we deployed recorders at predetermined points in a 3 × 3 grid with 50 m spacing at an open field site at the Pymatuning Laboratory of Ecology, Pennsylvania, USA. The positions of recorders were measured to within 20 cm precision using handheld GPS modules (Sparkfun RTK). AudioMoths with a GPS add-on were deployed on metal stakes ~1.5 m above the ground in plastic zip-lock bags with silicon desiccant packs for weatherproofing (SI Figure). All recorders were oriented in the same direction (North). Loudspeakers (PylePro PSBT85A 800 W loudspeakers) were deployed on the ground at predetermined known locations within the array, measured to within 20 cm precision using the handheld GPS modules. All speakers were oriented to face upwards to avoid directionality in playback. We adjusted the speaker volume based on a measurement of sound from 90 degrees off-axis (i.e., directly to the side of a speaker). We played a pink noise recording normalized to -3dBFS, and adjusted speaker volume to ensure a sound intensity of 88dBA (A-weighted dB SPL re 20 µPA) when measured at 1 m distance.

From the speakers we played pre-compiled audio files of recordings taken from Xeno-Canto.org (see Supplementary Table 2). Following^38^ we chose 6 focal species: Black-and-White Warbler *Mniotilta varia*, Black-throated blue warbler *Setophaga caerulescens*, Hooded Warbler *Setophaga citrina*, Black-throated Green Warbler *Setophaga virens*, Acadian Flycatcher *Empidonax virescens*, and Scarlet Tanager *Piranga olivacea*. As all focal species are summer breeding migrants at this site, we conducted the loudspeaker experiment in February when these species were not present. Bird positions were randomly selected from the 8 speaker positions, and species and individual identities were randomly selected from the set of recordings. We randomly generated 30 1-min soundscapes and concatenated these into a 30 min audio file for use during playback experiments.

### Forest deployment

**Fig. 2:**
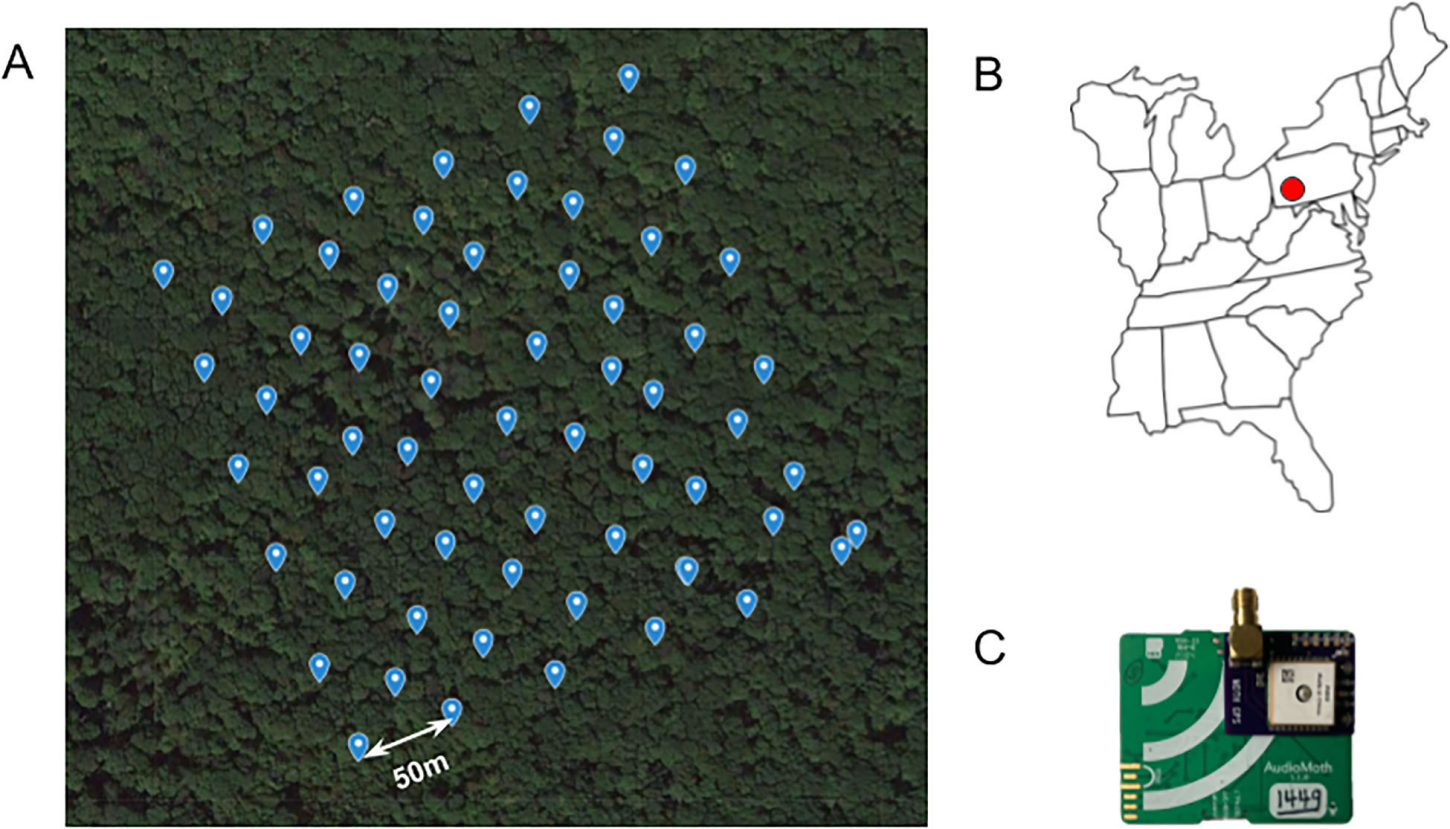
The forest deployment of recorders for acoustic localization. **A** A satellite photo of the site of the forest deployment, with the planned deployment positions of recorders noted with blue dots. Actual deployed positions were slightly off-grid. Image source: © CNES/Airbus, Google, Maxar Technologies 2025. **B** Geographical context (**C**) The Audiomoth-GPS hardware used for recording time-synchronized audio.

For the field experiment, we deployed an array of 61 recorders across 4 consecutive days at a site in Pennsylvania, USA, in mid-May 2022 (Fig. 2). We recorded for one hour a day beginning at 05:30 EDT (09:30 UTC) to capture the dawn chorus. 36 recorders were spaced 50 m apart in a 6 × 6 square grid, attached to a sapling trunk or small branch of a tree approximately 1.5 m above ground. The remaining 25 recorders were placed inside the center of each grid-square, deployed at approximately 2 m above ground. Recorders were strapped to small trunks or branches of trees (4–10 cm diameter) in plastic zip-lock bags with silicon desiccant packs for weatherproofing, and oriented in the same direction (North). The array covered approximately 6.25 hectares, part of State Game Lands 42, situated in the eastern deciduous forest of western Pennsylvania. The site is characterized by relatively flat terrain with minimal variation in elevation. Vegetation predominantly consists of mature deciduous forest with major canopy species including red oak (*Quercus rubra*), red maple (*Acer rubrum*), and hickory (*Carya*) spp. The understory varied from bare leaf litter to dense mountain laurel (*Kalmia latifolia*) thickets, with several open, fern-covered canopy gaps towards the center of the array.

We collected ground-truth data on the approximate locations of singing birds at the site of the array. A total of 7 spot-mapping surveys were conducted between 15 May and 6 June 2022 by CC and LMC. Two spot-mapping survey visits were conducted during dates the recorders were actively recording, and five further surveys were conducted in the 2 weeks after recorders had stopped recording. Expert surveyors conducted spot-mapping surveys following methods described in ref. ^39^, pp 42–64) and^40^. Spot-mapping involved walking a grid through the study site beginning shortly after dawn during peak singing activity, and recording visual or auditory observations of focal species on a paper map. Specific symbols were used to denote territorial behaviors or to mark occasions in which multiple individuals were detected at one time. Later, observations across all 7 visits were condensed onto a single map per species.

The 6 × 6 recorder grid was used to delineate spot-mapping routes. We placed a piece of bright flagging tape on each tree bearing a recorder to assist in determining the location of each bird observation. Each survey day, the surveyor would start in one corner of the grid and walk the length or width of the grid, then turn and walk back along the adjacent line in the grid, until all points in the grid had been visited. The combination of the corner chosen as the start point and direction chosen to walk at the beginning was never repeated throughout the survey period, such that each survey route was unique. Any observation of 5 focal species (Eastern Towhee *Pipilo erythrophthalmus*, Ovenbird *Seiurus aurocapilla*, American Redstart *Setophaga ruticilla*, Chestnut-sided Warbler *Setophaga pensylvanica,* and Hooded Warbler *Setophaga citrina*) was noted on spot-mapping maps, with one additional species (Common Yellowthroat, *Geothlypis trichas*) opportunistically added to the list during the first survey day. Spot mapping survey sheets were digitized, and only observations that were within the convex hull of the recorder array were used.

### Audio collection

We used open source AudioMoth acoustic recorders for data collection in both the loudspeaker experiment and forest deployment (Fig. 2C). We used a custom GPS add-on board, custom AudioMoth firmware and custom audio synchronization tools; though hardware, firmware and an audio synchronization tool officially supported by the AudioMoth manufacturer (Open Acoustics Devices) are now available (see https://www.openacousticdevices.info/audiomoth and https://www.openacousticdevices.info/gps-sync). AudioMoths recorded at 48 kHz, on medium gain.

To synchronize audio files for each date, the audio files were processed as described earlier. Overflows of the audio buffer on the recording hardware due to the non-deterministic latency of SD card writes have the potential to impact time-synchronization and corrupt audio - we designed our resampling process to identify buffer overflows, and none were detected in the recordings from either the loudspeaker test or forest deployment (see Supplementary Table 1).

### Convolutional Neural Network (CNN) training

For automated detection, we trained convolutional neural network (CNN) classifiers. We trained two separate CNNs for the two experiments, one for detection of the species used in the loudspeaker playback experiment, and another for the identification of species of particular interest in our study system. We used recordings from Xeno-Canto.org as training data for both CNNs. These recordings were then strongly labeled by expert annotators using Raven Pro^41^, who drew bounding boxes around every song of the focal species of the recording in the first minute of each file. We trained classifiers only for “song-type” vocalizations, so other vocalization types (e.g., calls) were ignored by expert annotators. The annotated audio files were used to produce training sets of 4 s audio segments labeled as positives (containing at least 1 s of song) or negative (not containing at least 1 s of song) for each species the CNN was trained to identify.

We trained CNNs using OpenSoundscape v0.10.1^29^. We used a ResNet50 architecture with weights pre-trained on ImageNet, with the classification head replaced by a final, fully connected layer with one output node for each species the CNN was trained to identify. See Supplementary Note 2 for CNN training details.

### CNN prediction

The trained CNNs were used to detect bird songs in the post-synchronization audio. CNNs produce continuous scores, one score for each class, for each discrete time-window. As we are in a ‘multilabel’ classification context where each discrete time-window may contain vocalizations of multiple species, we turn these scores into detections by applying thresholds for each class independently. Scores above the threshold are considered detections of the species, and scores below the threshold are considered non-detections. Where scores for multiple classes are above their respective thresholds, this time-window is considered a detection for all those classes. A logit-scale score of 0 was used as the threshold for both experiments.

### Localization framework

All the “tunable” parameters in the localization framework were kept the same for both loudspeaker and the forest deployment. See Table 1 and the earlier methods section for discussion of the parameters and design choices involved in the localization framework. We used a maximum inter-recorder detection distance (“max_receiver_dist”) of 150 m, a minimum cross-correlation score (“cc_threshold”) of 0.01, a minimum of 4 recorders (“min_n_receivers”) with TDOAs exceeding the cross-correlation threshold for localization to be attempted, and a maximum root-mean-square of the residuals (“residual_rms_threshold”) of 5 m for a point to be considered accurately localized. For DBScan clustering, we chose a distance of 5 m between points for them to be considered close neighbors, and a minimum number of three adjacent points. In both the loudspeaker test and forest experiment, position estimates were filtered to only those within the convex hull of the recorder array. See the SI for details on how these parameter values were chosen.

We quantitatively compared the spot-mapping observations with the point observations from the localization pipeline using Ripley’s Cross K statistic between the two point patterns using the Kcross function of the spatstat R package^42^, evaluated at a radius of 5 m. We simulated 5000 random toroidal shifts of the positions produced by the localization pipeline to construct a sampling distribution for Ripley’s K statistic under the null hypothesis that the spot mapping and localization position estimates are independent.

### Reporting summary

Further information on research design is available in the Nature Portfolio Reporting Summary linked to this article.

## Results

**Fig. 3:**
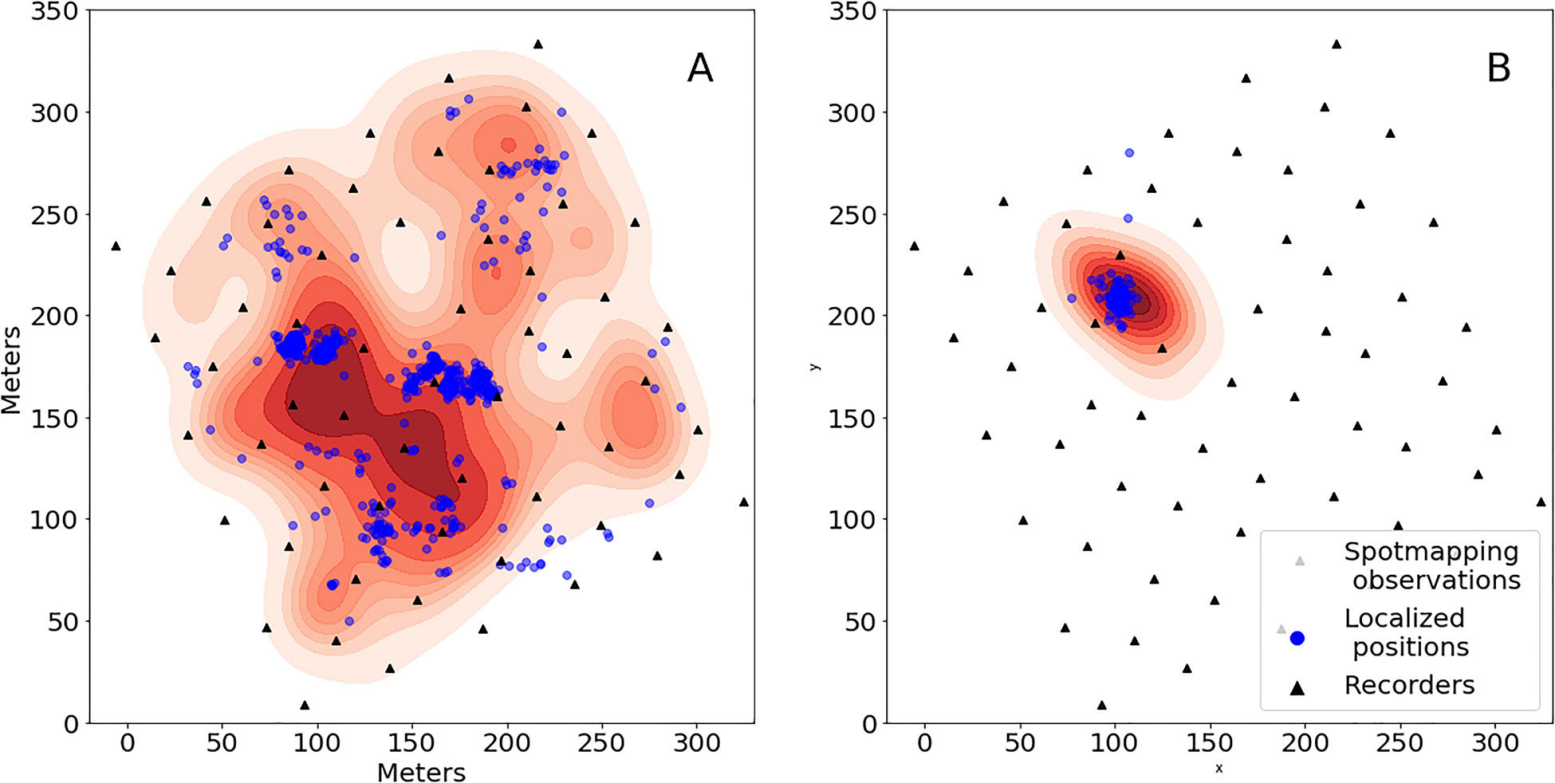
A comparison of patterns of observations within the array from spot mapping observations and automated acoustic localization. Spot mapping observations were smoothed with a kernel density and plotted as a heatmap along with the point localized positions produced by the localization framework for (**A**) Ovenbird (Seiurus aurocapilla) and **B** Common Yellowthroat (Geothlypis trichas). Point observations from spot mapping surveys were turned into kernel density estimated heatmaps to account for the fact that point observations were only approximate, and the acoustically localized points were overlaid on this heatmap.

In our loudspeaker experiment, the localization framework produced accurate sound source locations for bird vocalizations broadcast through the loudspeakers, as shown in Fig. 3. All species that were broadcast showed similar performance. Using localization parameters that aimed for spatial accuracy of 5 m or less, 99% of the position estimates were within 5 m of the loudspeaker broadcasting the vocalization. The mean position estimate error was 1.14 m. In addition to the accuracy of location estimates, an important aspect of the performance of the localization framework is its recall, i.e., what proportion of sound events were able to be localized. In the loudspeaker test, 34% of time-windows that contained at least 1 s of broadcast bird vocalization had a location estimated. This recall was reduced for the loudspeaker placed on the edge of the array, where no sounds broadcast during the 30 min test recording were successfully localized.

**Fig. 4:**
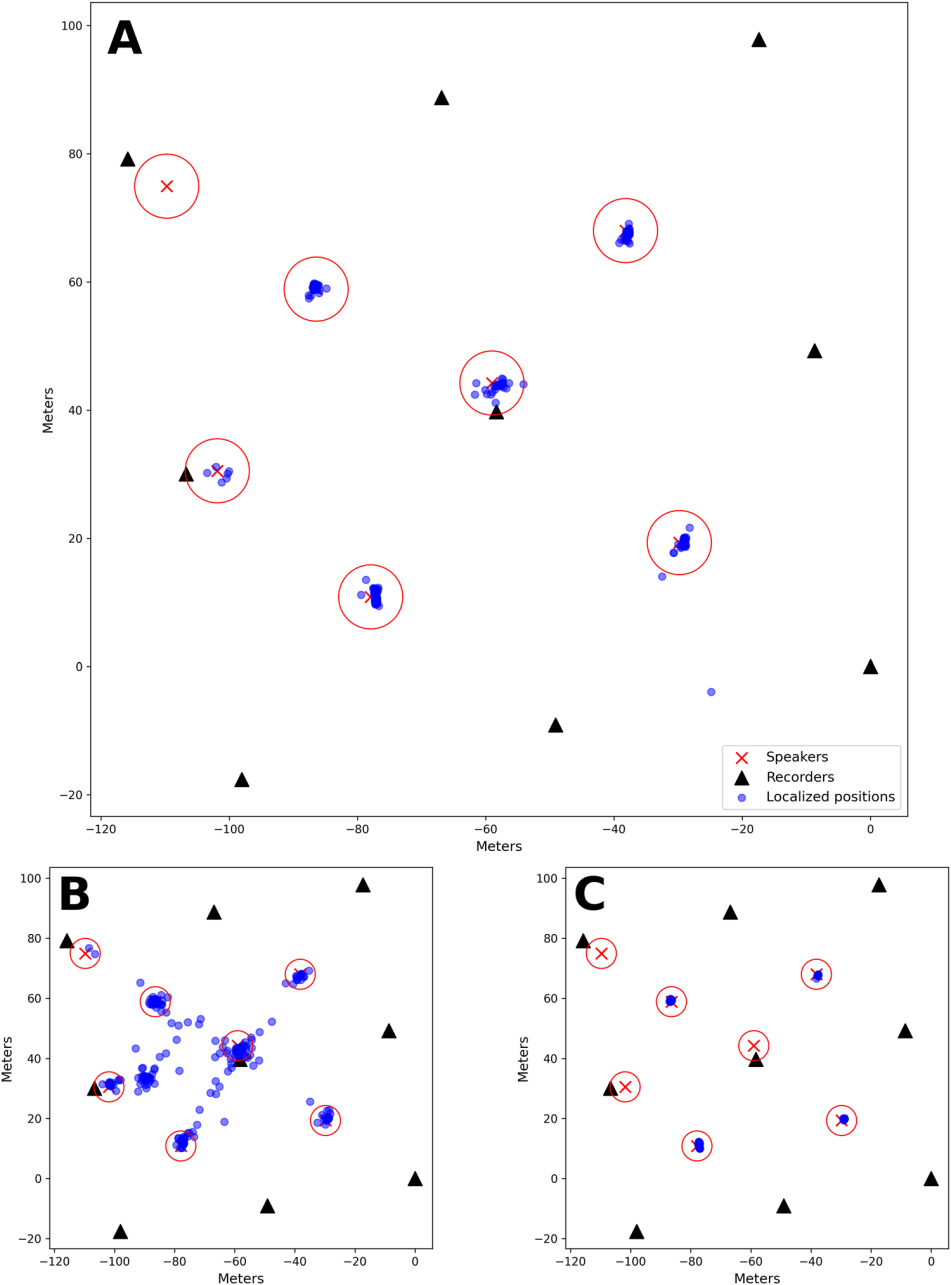
Localized positions produced by the automated localization framework on the loudspeaker test. Circles of 5 m radius are plotted in red around the true positions of the loudspeakers. The different panels show the effect of using parameters representing different levels of desired spatial accuracy in the automated localization framework. **A** Shows localization parameters used for all subsequent analyses of the field experiments. **B** Shows the localized positions using parameters that prioritize recall and lower the desired minimum spatial accuracy. **C** Shows the localized positions when using parameters that prioritize high spatial accuracy. See the SI for full details of the localization parameters used.

In the forest deployment, only Common Yellowthroat (*Geothlypis trichas*), Ovenbird (*Seiurus aurocapilla),* and American Redstart (*Setophaga ruticilla*) were observed within the localization array on all spot mapping visits. Spot mapping visits were mostly not simultaneous with the recording periods and spot mapping position observations were approximate, so direct comparison of point observations is not possible, however the overall patterns of observations across the array produced by both spot-mapping surveys and the automated acoustic localization framework were similar, as shown in Fig. 4. In the case of a species (Common Yellowthroat) that was noted in only one part of the array during all spot-mapping visits, the automated acoustic localization framework recapitulates very closely the observed spatial pattern. The localization framework produced localized singing positions for Common Yellowthroat that were tightly clustered on the same part of the array in which it was observed by spot mapping surveyors. The localization framework also produced similar patterns of observations for the species found at highest density, Ovenbird (*Seiurus aurocapilla)*, which had between 15 and 30 point observations per spot-mapping visit spread throughout the array.

## Discussion

Our results demonstrate that the fully automated localization framework can be used to produce accurate estimates of bird singing positions. By automating the process of detecting, localizing, and analyzing animal sounds within large localization arrays, this framework may significantly reduce the time and effort required for ecologists to collect precise coordinate data on animal locations. The also unlocks minimally invasive collection of fine spatial data on animal locations at large spatial and temporal scales. The acoustic localization framework is implemented in the Python package OpenSoundscape, and we present all code used in the associated GitHub repository and Zenodo. Further documentation is also available as part of the OpenSoundscape package (opensoundscape.org).

We verified the accuracy of location estimates of the framework in the speaker test, where 99% of estimated positions were within 5 m of the correct loudspeaker position. Vocalizations broadcast from one speaker at the edge of the test localization array were, however, not localized at all, suggesting that there is an important edge effect to be considered when using this localization framework. In the forest deployment experiment, the localization framework produced results consistent with territories identified in the spot-mapping surveys. In the simplest case of a conspicuous species represented by only a single individual found by spot-mapping surveys (Common Yellowthroat), the localization framework produces location estimates entirely consistent with the territory identified by surveyors. The observed cross K statistic between spot-mapping observations and localization pipeline estimates was greater than 99% of simulations that assumed independence of the two point patterns.

In a more challenging case of a species found at higher density (Ovenbird) within the array, the estimated locations output by the localization framework are also similar to patterns observed with spot-mapping surveys. The observed cross K statistic was greater than 93% of simulations that assumed independence, indicating substantial overlap between spot mapping and localization position estimates. Inferring territories from the spot-mapping point observations or acoustic localizations is challenging in the case of the more densely distributed Ovenbird. The localization framework however provides a considerable advantage in the spatio-temporal coverage possible compared to spot-mapping surveys, with the ability to collect spatial observations from across the entire array, for multiple hours.

It is important to note that the localization framework was not able to localize all calls broadcast from the loudspeakers. Of time-windows containing at least 1 s of bird vocalization broadcast, 34% were successfully localized. We were not able to quantify recall in the field experiment, however, it is likely that in more dense soundscapes, such as those of a dawn chorus, recall would be lower than that measured in the loudspeaker experiment. The low recall is a consequence of the multiple filtering steps applied throughout the localization pipeline that aid in discarding low-confidence location estimates. Compared to in-person surveys of avian positions and territories, we note that this relatively low recall is balanced by the ability to sample for much longer with autonomous recorders. The Audiomoth GPS units with current firmware can be used over multiple weeks for as much as 40 h of recording, though the exact battery life will depend on the firmware used and parameters such as sampling rate and recording schedule.

Edge effects are likely to reduce recall near the perimeter of a localization array, as fewer audio recorders are close enough to accurately detect and estimate TDOAs for a vocalization. This is a potential bias if using automated acoustic localization to investigate relative density of vocalization across a localization array. We suggest that this bias could be mitigated by excluding regions close to the edge of an array from further analyses, or by having a human surveyor count vocalizations in positions across the array and comparing these to automated localizations to estimate recall across the array.

A large part of the speed-up in analysis this framework enables relies on using an automated detection method. While we use a CNN, bespoke digital signal processing algorithms may perform excellently for specific sound types^43^. Pre-trained CNN classifiers for audio classification for a number of taxa are available, notably for the majority of global bird species through models like BirdNET^9^ and Perch^8^. Advances in deep learning for bioacoustics^7^ hold promise for the continued development of improved and novel classification methods. For taxa or vocalization types where existing classifiers are not available, hardware and software for training deep learning based classifiers are increasingly accessible. Automated detection systems make mistakes which can be quantified by their precision and recall. With low precision it is relatively simple to correct for false positives in the detection process by having an expert verify the presence of the detected vocalization in the audio. The automated pipeline can have an additional step of manual validation by displaying spectrograms aligned at the estimated TDOAs and having an expert verify both the species identity and the TDOA estimates. An automated detection system with low recall will, however, lead to few vocalizations that can be localized.

To apply the localization framework, a number of parameters must be chosen (see Table 1), and choices for these parameter values will depend on the research application and ecological contexts. Some of these parameters, such as the maximum detection distance over which two recorders are likely to have detected the same sound event, or the frequency range of sounds of interest, are easily interpretable. Other parameters, particularly those involved in the rejection of poorly localized position estimates, such as the minimum cross-correlation required for a TDOA to be used for localization, or the two parameters used by DBScan for clustering of multiple position estimates, may require careful consideration for optimization. We emphasize that while we explicitly outline all parameters so that future applications can optimize these, a user only needs to set the estimated detection distance and desired accuracy of location estimates to use the pipeline. There is necessarily a trade-off between the accuracy of sound-source estimates and the “recall”, or percentage of sound events which are able to be localized. To estimate these accuracy and recall values, deployments of localization arrays that use this or similar localization frameworks could include the broadcast of target sounds from within the array or the use of simultaneous human surveyors counting vocalizations to estimate accuracy and recall. We suggest future localization array deployments collect ground-truth data both for estimation of the pipeline’s performance and for optimizing the parameters of the localization pipeline.

Another consideration for future applications is that some acoustic signals and ecological contexts may be more challenging for applying acoustic localization. At the automated detection stage, louder sounds that present as visually distinct patterns in a spectrogram are likely to be well detected by a CNN. The directionality of a vocalization may also influence on which recorder a sound is detected on, with highly directional vocalizations being potentially harder to detect on enough recorders in a widely-spaced array, or leading to sets of TDOAs that may be more challenging to localize. The forest test results suggest accurate localization, however, we did not explicitly test the effect of directionality in our loudspeaker test.

We did not experience loss in the satellite signals affecting time-synchronization, however, satellite reception at a site is a consideration for field biologists deploying localization arrays. The time-synchronization process requires only the receipt of occasional GPS signals, and as such, perfect satellite reception is not needed throughout the recording period. However, during recorder deployment, the recorder positions must be precisely measured, as errors in relative positions may propagate through the localization framework. Measuring precise recorder coordinates during deployment may be challenging in densely forested ecosystems or sites with obstructed satellite signal.

Sound sources that are mobile and may move within a time-window used for detection, such as songbirds in flight (Dutilleux 2024), may also present a challenge for both time-delay estimation and localization from TDOAs. In some cases, these issues may be resolved by adjusting parameters such as the length of the time-window used for detection and localization, while others may require extensions of the framework.

Automated acoustic localization is of interest to ecologists working on many different vocally detectable taxa, including anurans, chiroptera, and orthoptera. However, applications of automated localization to these and other taxa is likely to pose specific challenges. Ultrasonic vocalizations like those of bats and some small mammals attenuate more rapidly, and hence require closely-spaced recorder arrays for detection and TDOA estimation. Highly stereotyped, periodic vocalizations found in dense choruses, such as those of some anurans and stridulating insects, may present a particular challenge. TDOA estimation can be error-prone for highly periodic vocalizations, particularly if the sound does not start or end within the time-window but is continuous throughout, as there are multiple plausible TDOAs separated by the period of the vocalization. In these cases, classifiers targeted to detect the onset of a new vocalization may be a practical solution, though the presence of a dense acoustic background of similar vocalizations may still prove a challenge to TDOA estimation.

We believe there are a number of areas for potential improvement, each of which could improve the performance of the localization framework at potential computational cost and added complexity. The estimation of time delays by cross-correlation is a key step in which errors may be introduced. To reduce error in TDOA estimation, we bandpass the signals to a species-specific frequency range that contains most of the energy in the vocalization and use a phase transform filter. However, particularly for noisy soundscapes, quiet vocalizations, continuous periodic vocalizations, or vocalizations that are short relative to the length of the time-window used for detection, TDOA estimation may be challenging. Where it is not possible to isolate signals of interest with a bandpass filter at specific frequency ranges, TDOA estimation may be particularly challenging. Noise reduction techniques^44^ and machine-learning based sound-source separation methods that can separate vocalizations overlapping in both time and frequency^45^ may improve localization performance. Additionally, methods for sound-source localization that do not rely on the two-stage process of estimating a TDOA and then using this TDOA for sound-source localization exist. Correlation accumulation methods, for example, may sum all the pairwise correlations across all recorders to estimate a coherent set of TDOAs, rather than estimating pairwise TDOAs between two recorders. Such approaches have been used in bioacoustic contexts^46,47^ and may be more robust to noisy soundscapes where TDOA estimation is error-prone, but would increase computational complexity.

Acoustic localization at scale is a minimally invasive monitoring method, and increasingly automated data collection enables a significant change in the kinds of ecological questions that can be investigated at scale. Quantifying territory sizes, habitat use within territories, singing behavior and individual movement are potential areas of research that would benefit from data collection enabled by a fully autonomous localization framework. Estimating abundance and delineating territories from acoustic data also becomes substantially more achievable with acoustic localization at scale. Vocalizations from known positions in territorial taxa also implies the identity of the animal, offering the potential for automated localization to associate recordings with the identity of the animal vocalizing: important data for training models for individual identification. By automating the acoustic localization process for passive acoustic monitoring data, our framework will support fine-scale investigations of these spatial processes.

## Supplementary information

Supplementary Information

Reporting Summary

## Data Availability

The audio data from the speaker test and scripts used for all the analyses are available on Zenodo^48^. Due to its large size, the audio from the field experiment will be made available upon reasonable request.

## Code availability

All analysis code is available in the GitHub repository: https://github.com/louisfh/loca_manuscript. The authors declare the following competing interests: Alex Rogers, Peter Prince, and Andy Hill are involved in the development of the AudioMoth recording hardware used in this study, which is available for sale to the public. All other authors declare no competing interests.
