# Supplementary material for: A fully automated framework for acoustic identification and localization of terrestrial wildlife at scale
Source: Commun Biol. 2026 May 9;9:975. doi: 10.1038/s42003-026-09949-5 (PMC13376177; doi:10.1038/s42003-026-09949-5)
Supplement: Supplementary file 1 — Supplementary Information [file 42003_2026_9949_MOESM1_ESM.pdf]

Supplementary Information

The code for the analysis can be found at the following github repository:
https://github.com/louisfh/loca_manuscript

Supplementary Note 1

Hardware details

Our GPS add-on board attaches to an AudioMoth v1.1. and v1.2.0 via a PMOD header connected to available GPIO pins. The board includes an Adafruit Ultimate GPS v3 receiver that was chosen due to its low cost and low power consumption. Before the start of a recording, a modified version of the AudioMoth firmware switches on the board and the GPS receiver. The receiver then obtains a satellite lock and begins sending a pulse per second (PPS) signal to the AudioMoth. The AudioMoth begins recording at the designated start time, with sampling proceeding based on the internal clock until the sampling period ends.

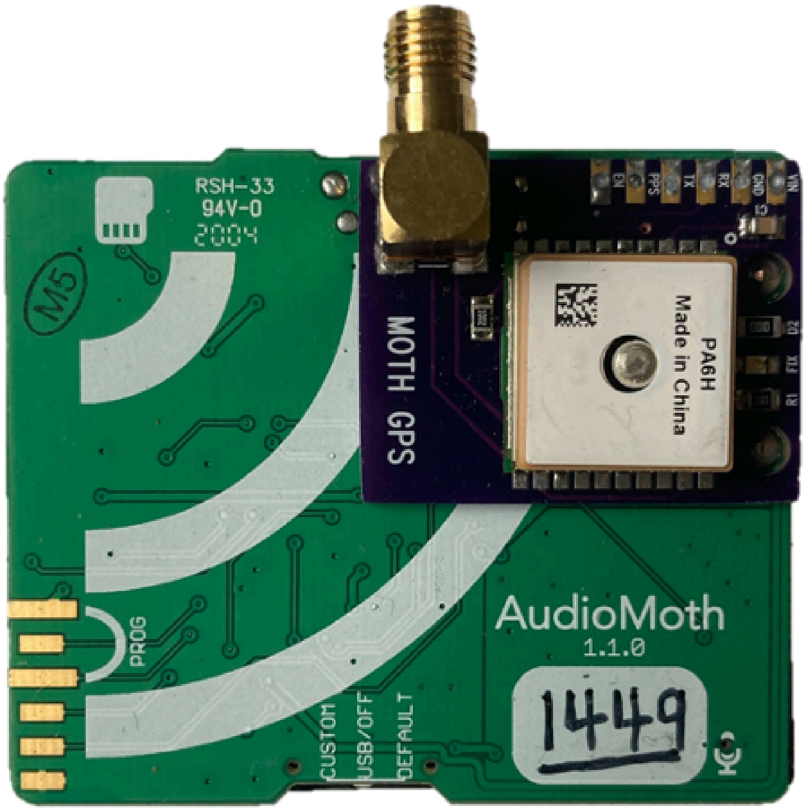

During the recording period, the GPS receiver remains active, and the recorder writes a metadata file to the AudioMoth's SD card as well as the audio file itself. The metadata file contains comma-separated values, with a new line written upon receipt of each pulse per second (PPS) signal. **See Supplementary Table 1** for the values written to the metadata file by the custom GPS AudioMoth firmware used. For the audio synchronization process the SAMPLES, LAST_RMC_GPS_TIME and PPS_NUMBER values were used. The BUFFERS_FILLED and BUFFERS_WRITTEN were used to check if buffer overflows had occurred during recording. We note that officially supported OAD firmware, called AudioMoth-GPS-Sync, is now available, along with two variants of GPS receiver boards.

Supplementary Table 1

Field	Notes
PPS_NUMBER	An integer counter corresponding to the number of PPS signals received since the device turned on (NOTE: in the files this often starts at ~207).
AUDIOMOTH_TIME	ISO 8601 timestamp of Audiomoth internal clock time.
SAMPLES	Number of samples written to WAV file since last PPS signal received.
TOTAL_SAMPLES	Total number of samples written to WAV file since recording started.
COUNTER	An integer [0-999] that measures how far through CPU 48Mhz clock cycle the microprocessor is. This could be used for sub-sample precision
BUFFERS_FILLED	Buffers received from the analog to digital converter.
BUFFERS_WRITTEN	Buffers written to SD card. If a buffer overflow occurs, this causes a +8 difference between BUFFERS_FILLED and BUFFERS_WRITTEN.
LAST_RMC_AUDIOMOTH_TIME	ISO 8601 timestamp
LAST_RMC_GPS_TIME	ISO 8601 timestamp
STATUS	
LAT_DEG	GPS location information
LAT_MIN	GPS location information
LAT_DIR	GPS location information
LONG_DEG	GPS location information
LONG_MIN	GPS location information
LONG_DIR	GPS location information

Supplementary Note 2

CNN training details

For both CNNs trained, spectrograms were cropped on the frequency axis to between 0 and 11025 Hz and scaled to 224x224 pixels to match the input size used by the ResNet50 architecture. During training, spectrograms were augmented with mixup of random samples

from Xeno-Canto recordings of other bird species; jitter of brightness, contrast and saturation chosen randomly from [-0.3,0.3]; and time and frequency masking by the random placement of 1-3 horizontal and 1-3 vertical bars of maximum width 22 pixels with uniform pixel intensity of the mean of the spectrogram pixel intensities. CNNs were trained for 50 epochs using an SGD optimizer, with an initial learning rate of 0.01, a scheduled learning rate decrease by a factor of 0.7 every 10 epochs, and a momentum of 0.9

Supplementary Table 2
The audio files used in the loudspeaker playback test.

xeno-canto.org Catalogue ID	Common Name	Latin
XC372010	Acadian Flycatcher	Empidonax virescens
XC416786	Acadian Flycatcher	Empidonax virescens
XC417181	Acadian Flycatcher	Empidonax virescens
XC420873	Acadian Flycatcher	Empidonax virescens
XC501241	Acadian Flycatcher	Empidonax virescens
XC500354	Black-and-white Warbler	Mniotilta varia
XC566119	Black-and-white Warbler	Mniotilta varia
XC568117	Black-and-white Warbler	Mniotilta varia
XC600303	Black-and-white Warbler	Mniotilta varia
XC613378	Black-and-white Warbler	Mniotilta varia
XC134502	Black-throated Blue Warbler	Setophaga caerulescens
XC186365	Black-throated Blue Warbler	Setophaga caerulescens
XC600512	Black-throated Blue Warbler	Setophaga caerulescens
XC600521	Black-throated Blue Warbler	Setophaga caerulescens
XC653335	Black-throated Blue Warbler	Setophaga caerulescens
XC173260	Black-throated Green Warbler	Setophaga virens
XC181473	Black-throated Green Warbler	Setophaga virens
XC389498	Black-throated Green Warbler	Setophaga virens
XC486860	Black-throated Green Warbler	Setophaga virens
XC299462	Hooded Warbler	Setophaga citrina
XC364660	Hooded Warbler	Setophaga citrina
XC408866	Hooded Warbler	Setophaga citrina
XC465133	Hooded Warbler	Setophaga citrina
XC565257	Hooded Warbler	Setophaga citrina
XC420841	Scarlet Tanager	Piranga olivacea
XC572720	Scarlet Tanager	Piranga olivacea

XC579106	Scarlet Tanager	Piranga olivacea
XC599850	Scarlet Tanager	Piranga olivacea
XC602554	Scarlet Tanager	Piranga olivacea

Supplementary Note 3

To quantify the effect of using different localization settings, we present the loudspeaker tests with three different sets of localization parameters. These results are presented in Figure 3 of the manuscript. The full set of localization parameters for each desired spatial accuracy are listed below

The localization parameters used

```
# lower desired minimum spatial accuracy
min_n_receivers = 4
max_receiver_dist = 150
cc_threshold = 0
rms_threshold = 20
cc_filter = "phat"

eps = 10
min_samples = 2
```

```
# Medium desired minimum spatial accuracy
min_n_receivers = 4
max_receiver_dist = 150
cc_threshold = 0.01
rms_threshold = 5
cc_filter = "phat"

eps = 5
min_samples = 3
```

```
# High desired spatial accuracy
min_n_receivers = 6
max_receiver_dist = 150
cc_threshold = 0.02
```

```
rms_threshold = 2
cc_filter = "phat"

eps = 3
min_samples = 5
```

Supplementary Note 4

To quantitatively test the agreement between the point observations of birds from spot mapping surveys and those produced by the automated acoustic localization, we calculated Ripley's K function between the two point patterns. We simulated 5000 random toroidal shifts of the point observations produced by automated localization, and the observed cross K statistics were greater than 99% of simulated cross K statistics for the Common Yellowthroat, and 93% of simulated cross K statistics for the Ovenbird.

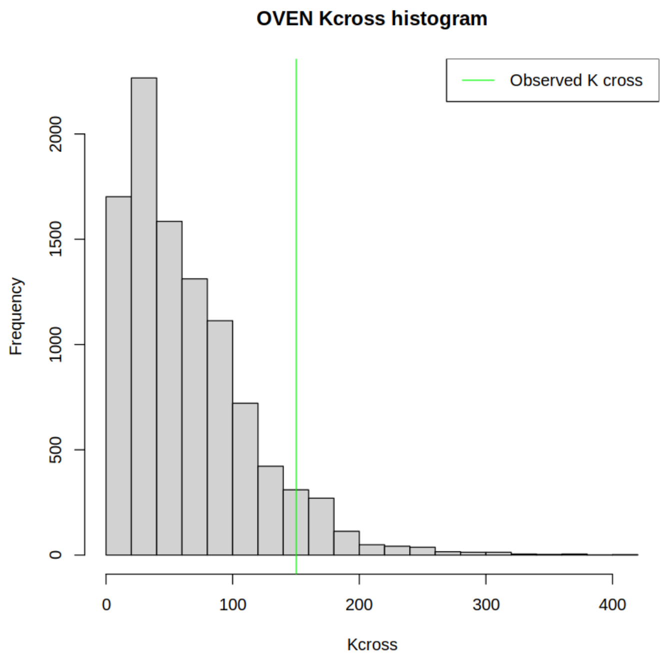

Supplementary Figure 1

The observed K cross statistic between Ovenbird localizations produced by automated localization and the observed surveyor locations is shown as a green line. The histogram shows the distribution of Ripley's K Cross statistics when the point observations produced by automated localization are subject to random toroidal shifts.

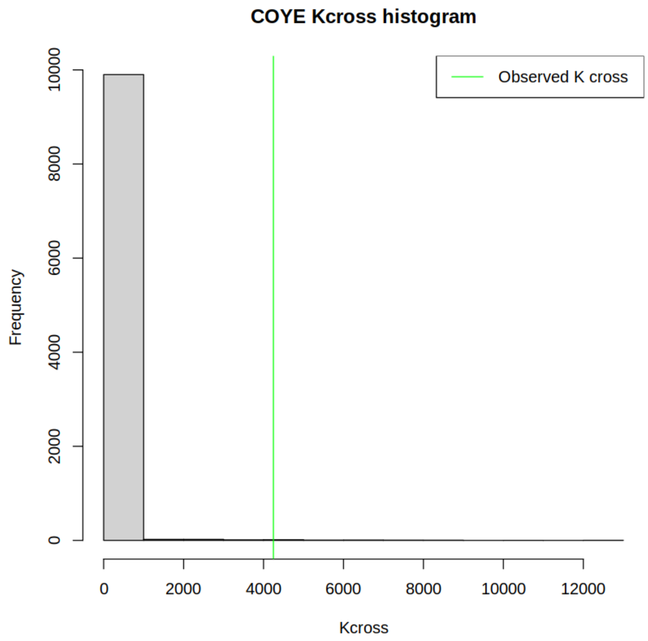

Supplementary Figure 2

The observed K cross statistic between Common Yellowthroad localizations produced by automated localization and the observed surveyor locations is shown as a green line. The histogram shows the distribution of Ripley’s K Cross statistics when the point observations produced by automated localization are subject to random toroidal shifts.
