# Supplementary material for: A fully automated framework for acoustic identification and localization of terrestrial wildlife at scale
Source: Commun Biol. 2026 May 9;9:975. doi: 10.1038/s42003-026-09949-5 (PMC13376177; doi:10.1038/s42003-026-09949-5)
Supplement: Supplementary file 2 — Reporting Summary [file 42003_2026_9949_MOESM2_ESM.pdf]

nature portfolio

Corresponding author(s): Louis Freeland-Haynes

Last updated by author(s): Nov 24, 2025

Reporting Summary

Nature Portfolio wishes to improve the reproducibility of the work that we publish. This form provides structure for consistency and transparency in reporting. For further information on Nature Portfolio policies, see our Editorial Policies and the Editorial Policy Checklist.

Statistics

For all statistical analyses, confirm that the following items are present in the figure legend, table legend, main text, or Methods section.

n/a	Confirmed	
☒	☐	The exact sample size (*n*) for each experimental group/condition, given as a discrete number and unit of measurement
☒	☐	A statement on whether measurements were taken from distinct samples or whether the same sample was measured repeatedly
☒	☐	The statistical test(s) used AND whether they are one- or two-sided<br>*Only common tests should be described solely by name; describe more complex techniques in the Methods section.*
☒	☐	A description of all covariates tested
☒	☐	A description of any assumptions or corrections, such as tests of normality and adjustment for multiple comparisons
☒	☐	A full description of the statistical parameters including central tendency (e.g. means) or other basic estimates (e.g. regression coefficient) AND variation (e.g. standard deviation) or associated estimates of uncertainty (e.g. confidence intervals)
☒	☐	For null hypothesis testing, the test statistic (e.g. *F*, *t*, *r*) with confidence intervals, effect sizes, degrees of freedom and *P* value noted<br>*Give P values as exact values whenever suitable.*
☒	☐	For Bayesian analysis, information on the choice of priors and Markov chain Monte Carlo settings
☒	☐	For hierarchical and complex designs, identification of the appropriate level for tests and full reporting of outcomes
☒	☐	Estimates of effect sizes (e.g. Cohen's *d*, Pearson's *r*), indicating how they were calculated

Our web collection on statistics for biologists contains articles on many of the points above.

Software and code

Policy information about availability of computer code

Data collection	Audiomoth recording units and their associated firmware were used to collect audio data.
Data analysis	Python v 3.10, R v4.3.1, spatstat v3.3.2, opensoundscape v0.10.2

For manuscripts utilizing custom algorithms or software that are central to the research but not yet described in published literature, software must be made available to editors and reviewers. We strongly encourage code deposition in a community repository (e.g. GitHub). See the Nature Portfolio guidelines for submitting code & software for further information.

Data

Policy information about availability of data

All manuscripts must include a data availability statement. This statement should provide the following information, where applicable:

- Accession codes, unique identifiers, or web links for publicly available datasets
- A description of any restrictions on data availability
- For clinical datasets or third party data, please ensure that the statement adheres to our policy

The audio data from the speaker test will be made available on Zenodo. Due to its large size, the audio from the field experiment will be made available upon request.

Research involving human participants, their data, or biological material

Policy information about studies with human participants or human data. See also policy information about sex, gender (identity/presentation), and sexual orientation and race, ethnicity and racism.

Reporting on sex and gender	n/a
Reporting on race, ethnicity, or other socially relevant groupings	n/a
Population characteristics	n/a
Recruitment	n/a
Ethics oversight	n/a

Note that full information on the approval of the study protocol must also be provided in the manuscript.

Field-specific reporting

Please select the one below that is the best fit for your research. If you are not sure, read the appropriate sections before making your selection.

☐ Life sciences ☐ Behavioural & social sciences ☒ Ecological, evolutionary & environmental sciences

For a reference copy of the document with all sections, see nature.com/documents/nr-reporting-summary-flat.pdf

Ecological, evolutionary & environmental sciences study design

All studies must disclose on these points even when the disclosure is negative.

Study description	We developed a novel acoustic localization pipeline. We demonstrated its performance on a loudspeaker test, and then applied it in a field study using a localization array in the eastern deciduous forest of PA, USA.
Research sample	Passively collected audio recordings of an avian community in a forested site in PA, USA.
Sampling strategy	Dates after the arrival of breeding migrants, the recording period was scheduled to be during peak vocal activity shortly after dawn.
Data collection	The audio data was recorded passively by autonomous recording units. The spot mapping surveys were conducted by Lauren Chronister and Chapin Czarnecki. They denoted the locations of birds on paper maps while walking transects along the recorder array.
Timing and spatial scale	*Indicate the start and stop dates of data collection, noting the frequency and periodicity of sampling and providing a rationale for these choices. If there is a gap between collection periods, state the dates for each sample cohort. Specify the spatial scale from which the data are taken*
Data exclusions	n/a
Reproducibility	No attempts were made to reproduce this study.
Randomization	n/a
Blinding	Blinding was not relevant to this observational study.

Did the study involve field work? ☒ Yes ☐ No

Field work, collection and transport

Field conditions	Field work was conducted in typical weather conditions (~25C, no strong wind, no storms forecast)
Location	Pennsylvania, USA,
Access & import/export	All appropriate permits for data collection were gathered from the Pennsylvania Game Commission.
Disturbance	The only disturbance was the presence of surveyors during spot mapping surveys, and during the deployment of recorders. Surveyors

Disturbance	made all efforts to minimize affecting the behavior of animals during their presence. Recorders were deployed by attaching them to trees, and were subsequently removed without damage to the vegetation.

Reporting for specific materials, systems and methods

We require information from authors about some types of materials, experimental systems and methods used in many studies. Here, indicate whether each material, system or method listed is relevant to your study. If you are not sure if a list item applies to your research, read the appropriate section before selecting a response.

Materials & experimental systems

n/a	Involved in the study	
☒	☐	Antibodies
☒	☐	Eukaryotic cell lines
☒	☐	Palaeontology and archaeology
☐	☒	Animals and other organisms
☒	☐	Clinical data
☒	☐	Dual use research of concern
☒	☐	Plants

Methods

n/a	Involved in the study	
☒	☐	ChIP-seq
☒	☐	Flow cytometry
☒	☐	MRI-based neuroimaging

Animals and other research organisms

Policy information about studies involving animals; ARRIVE guidelines recommended for reporting animal research, and Sex and Gender in Research

Laboratory animals	n/a
Wild animals	Wild birds of several species (notably Ovenbird and Common Yellowthroat) were observed by spot mapping surveyors. Surveyors made all efforts not to disturb these animals or alter their behavior.
Reporting on sex	n/a
Field-collected samples	n/a only audio recordings were collected.
Ethics oversight	n/a minimally invasive passive audio collection does not require ethics oversight.

Note that full information on the approval of the study protocol must also be provided in the manuscript.

Plants

Seed stocks	n/a
Novel plant genotypes	n/a
Authentication	n/a
